# Accurate Crystal Structure Prediction of New 2D Hybrid Organic–Inorganic Perovskites

**DOI:** 10.1021/jacs.4c06549

**Published:** 2024-09-30

**Authors:** Nima Karimitari, William J. Baldwin, Evan W. Muller, Zachary J. L. Bare, W. Joshua Kennedy, Gábor Csányi, Christopher Sutton

**Affiliations:** † Department of Chemistry and Biochemistry, University of South Carolina, Columbia, South Carolina 29208, United States; ‡ Department of Engineering, University of Cambridge, Cambridge CB2 1PZ, U.K.; ¶ UES, Inc., Beavercreek, Ohio 45432, United States; § Materials and Manufacturing Directorate, Air Force Research Laboratory, Wright-Patterson AFB, Dayton, Ohio 45433, United States

## Abstract

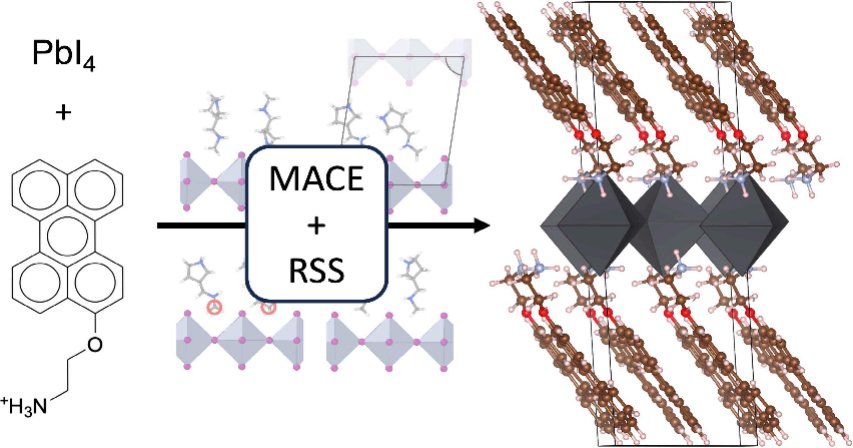

Low-dimensional hybrid organic–inorganic perovskites (HOIPs) are promising electronically active materials for light absorption and emission. The design space of HOIPs is extremely large, as a variety of organic cations can be combined with different inorganic frameworks. This not only allows for tunable electronic and mechanical properties but also necessitates the development of new tools for in silico high throughput analysis of candidate materials. In this work, we present an accurate, efficient, and widely applicable machine learning interatomic potential (MLIP) trained on 86 diverse experimentally reported HOIP materials. This MLIP was tested on 73 experimentally reported perovskite compositions and achieves a high accuracy, relative to density functional theory (DFT). We also introduce a novel random structure search algorithm designed for the crystal structure prediction of 2D HOIPs. The combination of MLIP and the structure search algorithm reliably recovers the crystal structure of 14 known 2D perovskites by specifying only the organic molecule and inorganic cation/halide. Performing this crystal structure search with ab initio methods would be computationally prohibitive but is relatively inexpensive with the MLIP. Finally, the developed procedure is used to predict the structure of a totally new HOIP with cation (*cis*-1,3-cyclohexanediamine). Subsequently, the new compound was synthesized and characterized, which matches the predicted structure, confirming the accuracy of our method. This capability will enable the efficient and accurate screening of thousands of combinations of organic cations and inorganic layers for further investigation.

## 1 Introduction

**Figure 1:**
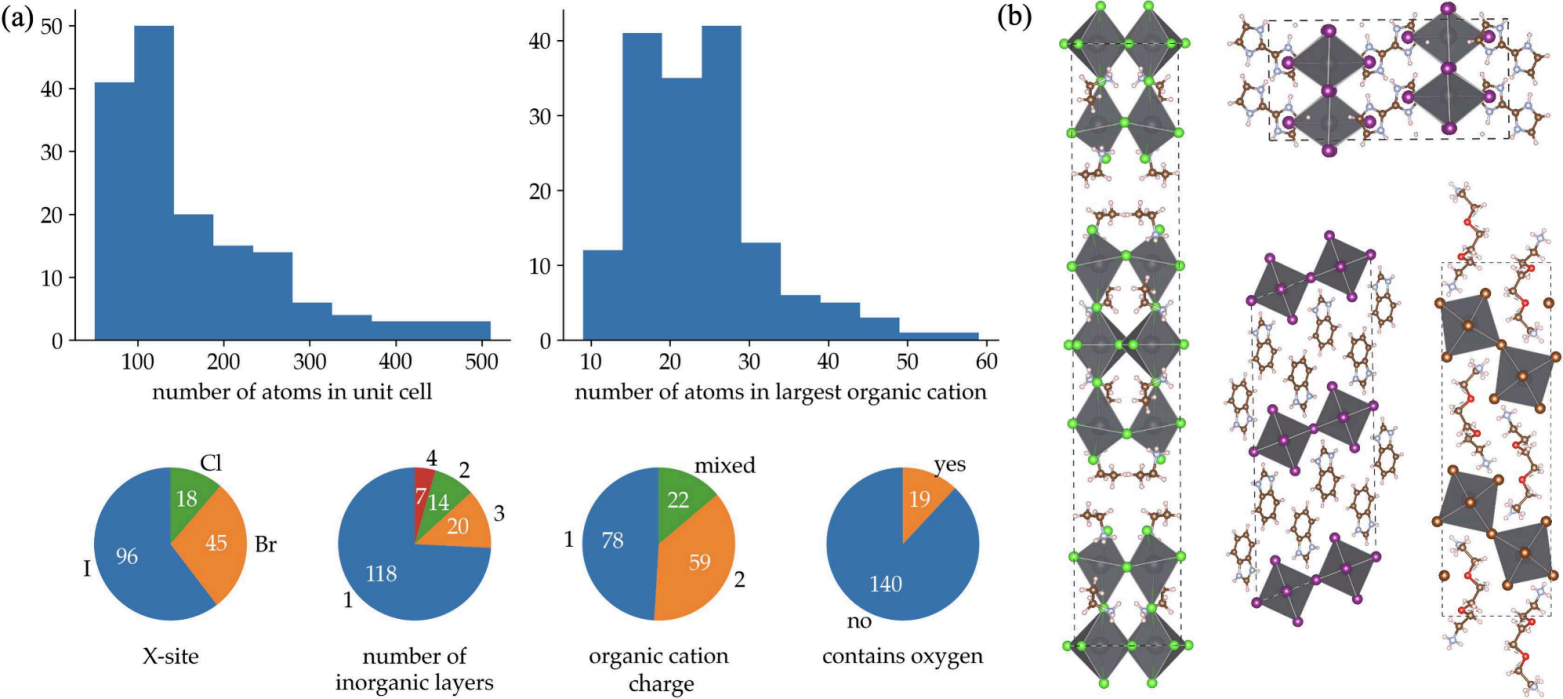
(a) Overview of the samples in the compiled 2D HOIP data set. Note that some structures have multiple organic cations, but the upper right histogram shows only the size of the largest cation in each structure. (b) Examples of 2D HOIP structures in the data set.

Hybrid organic–inorganic perovskites (HOIPs) belong to a broad category of materials, generally represented by the chemical formula ABX_3_. The B-site and X-site ions form a network of corner-sharing BX_6_ octahedra. Although the A-site can be a large inorganic cation, such as cesium, using an organic cation has proved extremely successful, resulting in the development of state of the art solution-processed optoelectronic materials.^1^ Provided that the organic cation is small, the typical perovskite structure is retained. For larger cations, however, the network of corner sharing octahedra is disrupted, leading to “low dimensional” structures such as one-dimensional chains or two-dimensional sheets of octahedra (see Figure 1b).

Two-dimensional HOIPs are formed when the organic cations separate the inorganic layers in the (100), (110) or (111) direction, giving the modified general formula A_*m*_^′^A_n_B_*n*_X_3*n*+1_. The constants *n* and *m* determine the number of connected inorganic layers and the charge of the organic cation. They are further categorized into two main types: Dion–Jacobson (DJ)^2^ with *m* = 1 (one sheet of interlayer cations with +2 charge) and Ruddlesden–Popper (RP)^3,4^ with *m* = 2 (two sheets of cations with +1 charge).^5^ Two dimensional HOIPs have the advantages of enhanced stability under ambient conditions and structural tunability. This makes them promising candidates for applications in photoluminescence (PL), photovoltaics, photodetection, and light emitting diodes (LEDs).^6−9^

Due to the breadth of the design space of 2D (as well as 1D and 0D) perovskites, in silico property screening is desirable. However, in order to calculate properties with ab initio electronic structure methods, one first must know the crystal structure. A similar task has been tackled in the field of organic crystal structure prediction (CSP): typically, CSP methods involve generating many hundreds or thousands of candidate structures, and selecting the lowest energy structures using an empirical force field.^10^ For general inorganic crystals, several algorithms including Random Structure Search (RSS),^11^ minima-hopping (MH),^12,13^ evolutionary algorithms (EA),^14^ basin-hopping (BH)^15^ or a mixture of these methods^16^ have been successfully used for unit cells of up to a few hundreds of atoms.

A particular difficulty in crystal structure prediction of 2D HOIPs is that they can have extremely large unit cells containing up to 1000 atoms. Furthermore, they are structurally complex (see Figure 1) with the organic molecules having many potentially quite flexible degrees of freedom, and can form many different phases.^17^ Direct Density Functional Theory (DFT) geometry relaxations and molecular dynamics simulations are therefore prohibitively expensive. Alternatively, empirical force fields that are accurate across the desired range of chemical interactions do not presently exist. Attempts have been made to overcome these issues: Namely, Ovčar et al.^18^ used an approach which combined empirical potentials and DFT to perform MH^12^ and therefore structure prediction of 2D HOIPs. This method performed well for a limited number of test cases, but it is unclear how it could scale to the full design space of these materials.

Machine-learned interatomic potentials (MLIPs) are an accurate and efficient alternative to DFT or empirical force fields.^19−22^ MLIPs can be trained to predict the potential energy of a configuration of atoms directly from the atomic coordinates, allowing for simulations of hundreds of thousands of atoms at DFT accuracy.^23^ Many MLIP architectures have been developed in recent years. Key developments in this area have been the focus on atom-centered energy contributions enabling linear scaling models, the incorporation of physical symmetries into model architectures^24−26^ and efficient construction of many-body representations of atomic environments.^27−29^ Furthermore, the introduction of graph-based MLIPs has greatly improved accuracy and transferability of these models.^30−32^ MLIPs have already been used to perform structure prediction for large scale screening tasks, including a computational study searching for novel stable inorganic materials.^33^ MLIPs have also been used in combination with EA in studying multicomponent inorganic compounds,^34,35^ or with BH in studying global optimization of gold clusters.^36^

In this work, the MACE^37^ message passing architecture was used to build a transferable MLIP for HOIPs. MACE is a graph tensor network which constructs many-body equivariant messages at each node (nodes correspond to atoms in this case) via the atomic cluster expansion,^28^ which are then passed onto neighboring nodes. The architecture has been shown to be accurate, efficient and transferable,^38^ and has recently been used to create a state of the art ML organic force field^39^ and a “foundation model” for materials chemistry.^40^ The model in this work is fitted to data collected from several publicly available databases of experimentally reported HOIPs. Starting from structures reported in these databases, an extensive training data set was generated by running an active learning protocol based on molecular dynamics. Collected configurations were labeled with DFT calculations. The final model achieves excellent accuracy across an independent set of perovskites with unseen compositions that are taken from the same sources.

To use the model effectively, we present a random structure search (RSS) procedure designed for 2D HOIPs and show that the trained MLIP accurately captures the complex potential energy landscape encountered during a random structure search task. The constrained RSS process we propose relies on a novel way to sample the space of 2D HOIP structures that is broadly applicable and efficient. The combination of the structure searching algorithm and the MACE model is an accurate and fast structure prediction tool. This is shown by “discovering” the ground state structure for a set of experimentally reported HOIPs not seen by the model during training, given only the most basic information on the perovskite, the identity of the organic cation, and the composition of the inorganic layer.

Finally, we predict the crystal structure of a previously unreported 2D HOIP material. We then synthesized this material and verified that the structure agrees with our prediction. Interestingly, in addition to the ground state structure, the MLIP-based structure searching algorithm reveals a large number of competing low energy minima, with subtly different orientations and stacking patterns of the organic cation. Therefore, due to the high degree of similarity between these structures, an accurate and efficient search tool offers many insights beyond just prediction of the ground state.

## 2 Data Set Construction

A data set was compiled from three sources: The 2D perovskites database of the laboratory of new materials for solar energy (NMSE),^41^ the Cambridge Structural Database,^42^ and a recent research article by Tremblay et al. reporting numerous 2D HOIP structures.^43^

The occurrence of different chemical elements and structural features in these sources was quite nonuniform. Several simplifying restrictions have therefore been placed on the scope of our model. First, the set of chemical elements considered for the inorganic layer was restricted to only include Pb,I, Br, and Cl. As a result, the resulting MLIP can be applied to only Pb-based perovskites with X = I, Br, or Cl. Furthermore, we restricted the composition of the organic cation to include only C, H, N and O. These restrictions were imposed due to the occurrence of different chemical elements in the available 2D HOIP data sets: of the structures we collected, more than 80% were lead-based, and the majority contained only C, H, and N elements in the organic cation. Applying these filters resulted in an initial data set of 159 experimentally reported structures. Figure 1a presents an overview of the data set including the number of atoms in the unit cell and organic cation, as well as a breakdown of the elements present at the X-site, number of inorganic layers, organic cation charge, and whether the organic cation contains oxygen. In brief, number of atoms per unit cell ranges from 50 to 510 atoms, with an average of 165 atoms per unit cell. Four representative example structures are shown in Figure 1b to illustrate the diversity of the perovskites that are included.

## 3 Results and Discussion

### 3.1 Model Development and Performance

#### 3.1.1 Active Learning for Data Set Expansion

In the following, *composition* will refer to a perovskite as determined by the chemical formula and the experimentally reported unit cell, while *configuration* will refer to a specific nonequilibrium set of atomic positions, for which one could compute a reference energy using DFT. The data set described above serves as a starting point for fitting an MLIP. In practice, however, fitting accurate and stable models requires a database with many nonequilibrium configurations for each target composition or phase. One popular method for database construction is to sample configurations from molecular dynamics trajectories. In this study, a different approach is taken in which a database of reference configurations is grown iteratively in an active learning procedure.^44,45^

Before beginning the active learning procedure, the overall data set of 159 experimentally reported compounds was first divided in two by randomly sampling 86 perovskites to form the core of the training set. The remaining test set compositions were used to assess the transferability of the final model to unseen perovskites.

The key principle of active learning is to use a model that can estimate the uncertainty of its own prediction on a given configuration. If this estimate is reliable, one can search for configurations on which the model is uncertain and add only these configurations to the data set. Several methods exist for constructing MLIPs with an built-in measure of prediction uncertainty. For MACE, the uncertainty estimate can be obtained using several independently trained models with the same hyperparameters but with a different random initialization of weights (referred to as a committee) for a given training set. The training set is identical for each committee members. In this work, we use 3 independently trained models to calculate uncertainties. On a new configuration, the disagreement between the committee members can be treated as an uncertainty estimate. As will be shown below, we find 3 members to be sufficient in identifying samples that have large disagreement in force predictions. In addition, there is no evidence that having more members and more precise uncertainty measurement leads to faster convergence of the activate learning procedure.

With this method for assessing the uncertainty of a model, the active learning procedure is as follows:

1. Given an initial data set of configurations, calculate reference energies and atomic forces using DFT. Fit a committee of 3 MACE models on this data set.
2. For each composition in the training set, run an MD simulation starting from the experimentally reported structure and using the average of the force predictions of the committee members to propagate the dynamics. At each time step, test the uncertainty of the potential by calculating the disagreement in the prediction of the atomic forces between the committee members.
3. If the *relative* force uncertainty of any atom, defined as the standard deviation of the committee force predictions divided by the mean of the forces, is larger than a specific threshold (in our study this threshold is to 0.2, see also section 9.2) the MD simulation is terminated. DFT energy and forces are calculated for the configuration for which the uncertainty exceeded the threshold, and added to the training set. If the uncertainty does not exceed the threshold within 10 ps, terminate the MD and do not collect any new configurations.
4. Refit the committee of models with the expanded data set. It is expected that the configurations where the models previously disagreed are now well described with low uncertainty.
5. Repeat steps 2–5 until no new configurations are collected for any of the compositions.

In each cycle of active learning, we ran committee MD at 300 K for each of the 86 compositions in the training set. Additional configurations are therefore collected at a rate of 86 per cycle if the uncertainties for all compositions exceed the threshold. However, as the data set grows, many compositions quickly become well described and do not trigger new DFT calculations, resulting in few new training configurations per cycle. For this reason, in later cycles, step 3 was repeated 10 times for each training set composition before retraining the model. The final potential is fitted once all the unique perovskite compositions in the training set are stable, meaning that in 10 independent MD simulations lasting 10 ps each, the 0.2 relative force uncertainty threshold is not exceeded. Additional details on the active learning procedure are given in section 9.2

**Figure 2:**
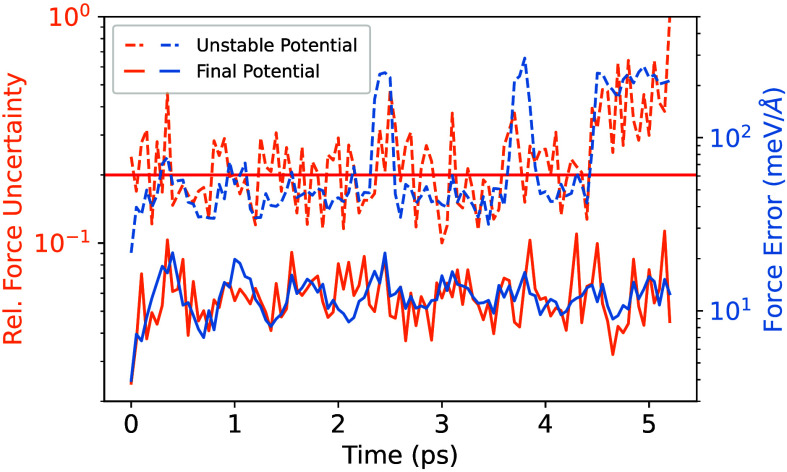
Relative force uncertainty and actual force error for one HOIP as a function of time during an MD simulation. The unstable potential (dashed lines) occasionally exceeds the relative force uncertainty threshold (red solid line at 0.2) with actual force errors as large as 100 meV/Å, while the final potential remains far below the threshold with force errors fluctuating between 10 to 20 meV/Å.

Key to this method is the reliability of the uncertainty measure. An example of the evolution of the uncertainty measure during a committee MD simulation is shown in Figure 2. To assess the uncertainty measure, all configurations in the trajectory were evaluated with DFT, and the actual force error made by the model at each time step is also shown. Two models from different stages in the active learning procedure are shown: an “unstable” model from an early point in the active learning process and the final model.

For the unstable potential, the uncertainty exceeds the threshold (the solid red line) at multiple instances and eventually increases to 1.0 implying total uncertainty in force predictions. By contrast, the final potential has both a consistently lower uncertainty and a lower force error. The key result shown in Figure 2 is that the difference in force error between the final model and unstable model is clearly reflected in the estimated uncertainty. Also important is that the spikes in the force error of the unstable model closely correlate with the spikes in the relative force uncertainty.

In general, the highest uncertainty occurs for atomic configurations that are less represented in the training set. In particular, there are 61 unique organic cations in the 86 compositions of the training data set, while there are just 7 types of inorganic layer. Therefore, the highest force uncertainty typically occurred on organic cations.

#### 3.1.2 Final Model Performance

**Figure 3:**
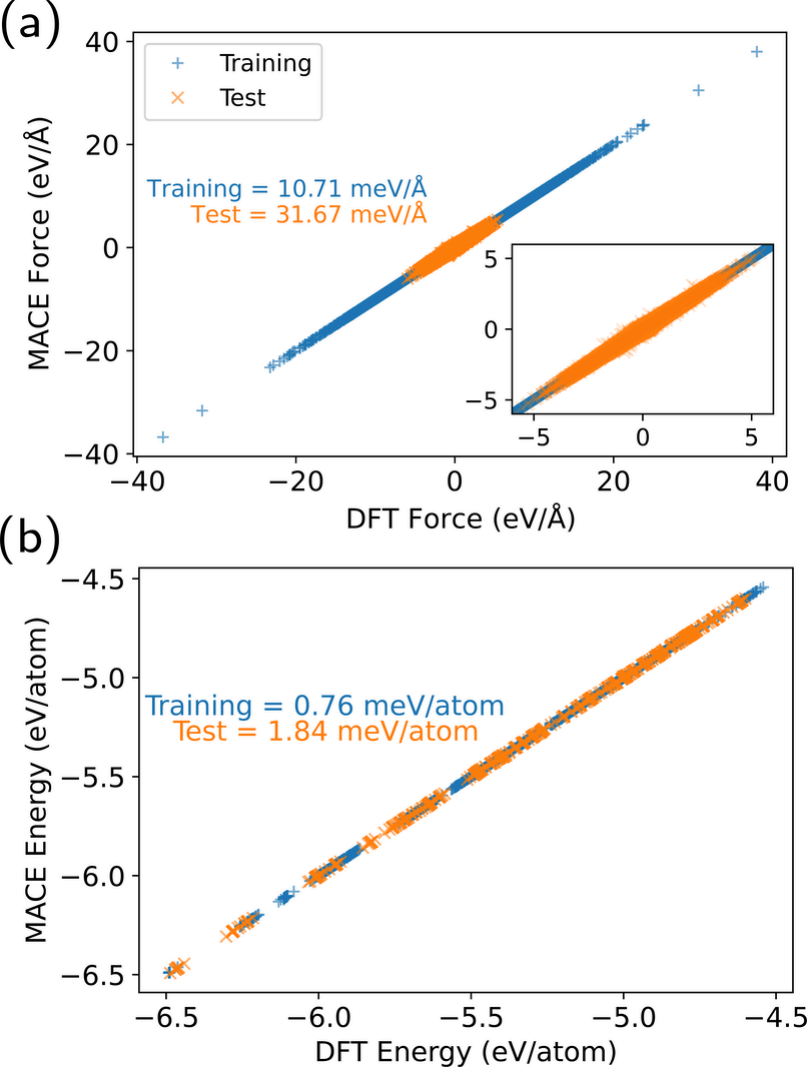
Parity plot of (a) forces, and (b) energy (per atom) for training and test set samples.

In total, 18 cycles of active learning were performed. The final training data set contains 2457 configurations. To test the final potential, MD simulations of 73 unseen test set compositions were run for 10 ps, and samples were taken every 1 ps. The energy and force predictions for all the training and test samples are shown in Figure 3. The root-mean-square error (RMSE) of the training (test) data set for energy and forces are 0.76 (1.84) meV/atom and 10.7 (31.7) meV/Å, respectively. In addition, the errors categorized based on the halide atoms are shown in Table 1.

**Table 1:** Energy and Force Errors for Seen and Unseen Configurations Categorized Based on the Halides

	Seen Compositions		Unseen Compositions	
	Energy (meV/atom)	Forces (meV/Å)	Energy (meV/atom)	Forces (meV/Å)
Cl	0.86	9.25	1.86	30.4
I	0.74	10.96	1.34	29.88
Br	0.78	10.39	2.12	48.53
				
Total	0.76	10.71	1.84	31.67

### 3.2 Relaxation of Experimentally Reported Structures

Experimentally reported structures are typically close to the global minima of the potential energy surface. For the trained MACE model to be useful for structure searching, it must relax these structures to the same local minima as those obtained by a DFT geometry relaxation. To assess whether this is the case, we considered 117 perovskite compositions in the data set that have less than 200 atoms, with 58 from the training set and 59 from the test set. For all of these compositions, the experimentally reported structure was relaxed independently with DFT and the MLIP, until the forces were less than 10 meV/Å.

**Figure 4:**
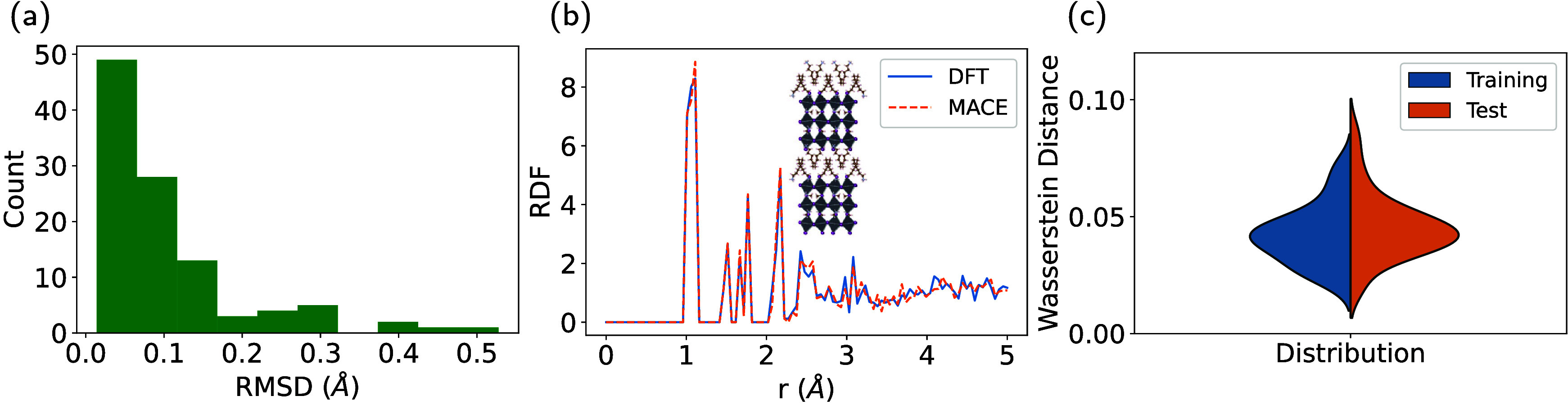
Evaluating the MLIP for geometry relaxations of experimentally reported structures. (a) Histogram of the RMSD between the DFT relaxed and the MLIP relaxed structures for the entire data set. (b) Comparison of the total RDF for a test set structure after relaxing with DFT and the MLIP. (c) The distribution of the Wasserstein distance between the RDFs given by the DFT relaxed structure and MLIP-relaxed structure for 117 unique HOIPs in the training and test set.

One way to quantify the difference between the MLIP and DFT relaxed structures is to measure the root-mean-square displacement (RMSD) of the atoms between the two structures. The distribution of the RMSD for all 117 compositions is shown in Figure 4a. For the majority of the samples, the RMSD is less than 0.1 Å. Several outliers are present with larger RMSDs on the order of 0.3–0.5 Å. These outliers generally correspond to cases in which long, flexible organic molecules move slightly with respect to each other.

The independently obtained DFT and MLIP relaxed structures can also be compared using the total radial distribution function (RDF), which contains information about the bond lengths, intermolecular distances, and organic–inorganic distances in the structure. A comparison between the RDFs of an MLIP and DFT relaxed structure is shown in Figure 4b. For *r* < 3 Å, which mostly corresponds to the intramolecular bond distances, the differences between MLIP and DFT structures are negligible. For *r* > 3 Å, which contains both the intermolecular distances and inorganic bonds, some differences are apparent; however, the structures relaxed with MLIP and DFT share many of the larger features.

To quantify the difference between the RDFs of MLIP and DFT relaxed structures, we used the first Wasserstein distance (also referred to as the earth mover’s distance) between these two distributions, which calculates the least amount work required to change one distribution to the other.^46^ A histogram of the Wasserstein distances for 63 randomly selected compositions in the training and test sets is shown in Figure 4c. One can see that the final MLIP performs similarly for both training and test sets using this metric.

### 3.3 High Throughput Structure Prediction for New HOIPs

Generally, calculating properties of known perovskite structures with ab initio methods is expensive but not impossible. On the other hand, crystal structure prediction of many new compositions is prohibitively costly and unfeasible for high throughput screening, particularly for structures with large unit cells. This is because crystal structure prediction protocols typically involve a very large number of either geometry relaxations or single point evaluations to predict the structure of just one chemical composition.

In particular, organic crystal structure prediction involves first generating many (thousands) of candidate crystal structures by enumerating key variables, such as space groups, and employing heuristics. Single point evaluations with empirical force fields are used to select good candidates, based on lowest potential energy.^10^

Ab initio random structure search (AIRSS) is another approach that has been explored,^11^ particularly for inorganic crystals. In this approach, crystal structures are determined by first guessing random positions of atoms within the unit cell, followed by geometry relaxations with DFT. Again, the lowest energy structure is chosen as the most probable structure. AIRSS has been employed successfully to find ground state structures of materials, molecules and features such as defects.^11^ This process is powerful but limited to small unit cells due to the *N*^3^ scaling of DFT, where *N* is the number of electrons.

In the following, we introduce a simple structure search procedure inspired by these ideas, which is appropriate for 2D HOIPs.

#### 3.3.1 RSS Procedure for 2D HOIPs

**Figure 5:**
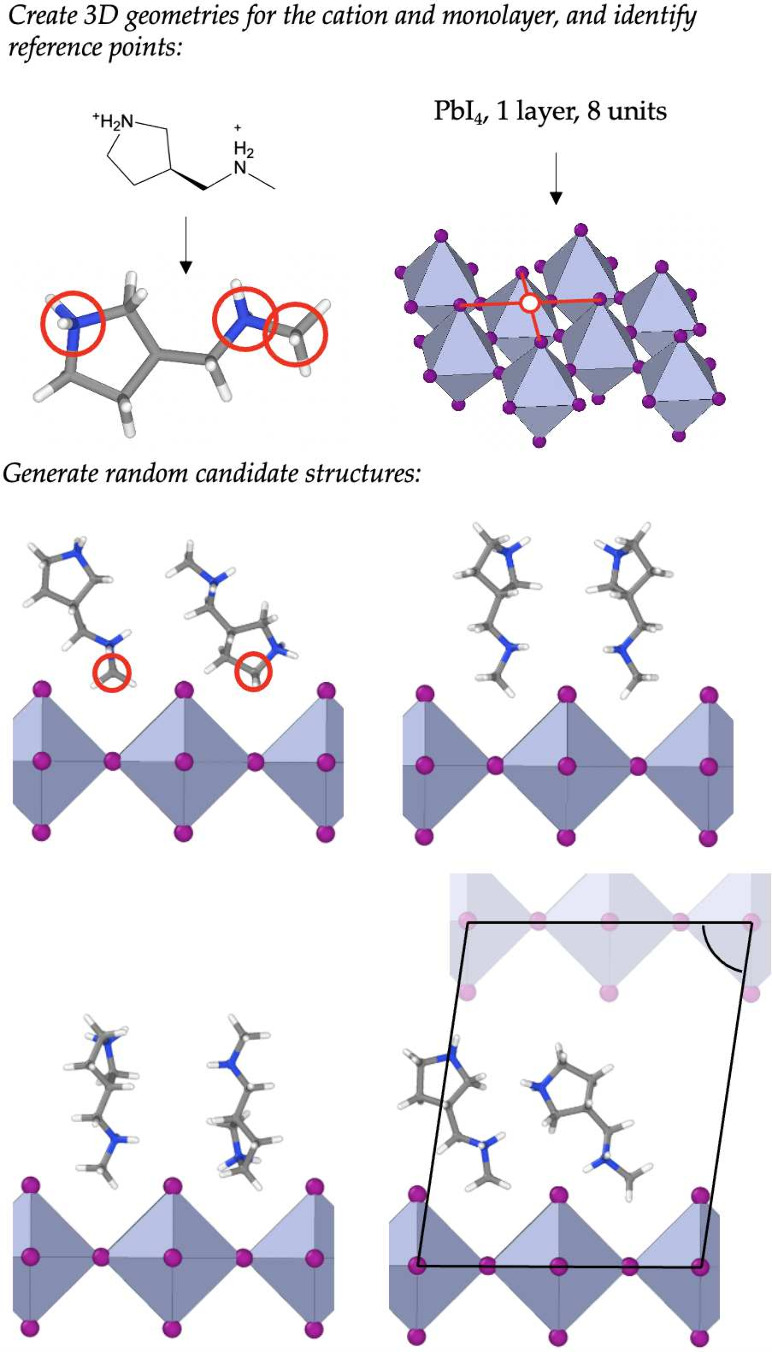
Overview of the structure generation algorithm for creating initial guesses for the RSS process. To make the figure more readable, the unit cell is only shown for one of the four candidate structures in the lower panel.

Our proposed structure searching workflow is summarized as follows: For a given organic cation and inorganic layer, a fixed number of candidate structures, which cover the space of feasible molecular and atomic arrangements. The geometry of all structures is then relaxed to a local minimum using the MLIP and the lowest potential energy structure is declared as the most probable crystal structure. The process for generating random candidate structures is key, and a scheme was designed based on several simple heuristics. The steps are summarized as follows and shown visually in Figure 5:

1. The starting information is the identity of the organic cation, the choice of halide, and the desired size of the unit cell. The size is determined by the number of organic/inorganic layers, and the number of octahedra per layer in the unit cell.
2. For the given composition, construct the 3D geometry of the organic cation (enumerating or sampling conformers if necessary). Also construct the untilted, strain-free inorganic layer from lead and the chosen halide. This determines the periodicity of the system in the in-plane directions.
3. Identify “reference points” on the cation and the inorganic layer. On the cation, reference points are defined as formally charged atoms or salient atoms. On the inorganic layer, the reference points are chosen to be the midpoints between protruding halides, as shown in Figure 5.
4. Based on the charge of the organic cation, determine the number of cations per layer required for charge neutrality. For each organic cation in the unit cell, randomly generate a set of reflections and rotations, subject to some constraints, to apply to the organic cation. Subsequently, place the transformed cations onto the inorganic layer by pairing reference points on the two geometries.
5. Check for any intersections between cations, or intersections of cations with the inorganic layer. Discard samples for which these components intersect one another.
6. Fix the lattice constant in the out-of-plane direction to remove most of the vacuum region from the cell, including some amount of shear of the unit cell. If more than one inorganic/organic layer per unit cell is desired, repeat the above procedure and stack the resulting geometries.

This process gives structures that sample the configuration space well but can contain high energy features, such as regions of vacuum or atoms at energetically unfavorable separations. Crucially, the configurations are sufficiently sensible that geometry relaxation leads to reasonable structures.

A python package was written to implement this algorithm which is available at https://github.com/WillBaldwin0/LDHP-builder. The algorithm is specific to 2D corner-sharing HOIPs, since it relies on heuristics when placing molecules onto the inorganic layer. In practice, it was found that these heuristics perform remarkably well. The heuristics for identifying reference points and symmetry constraints are important for efficiency and generality of the scheme; further details are provided in section 5.4.

In a recent work by Ovčar et al.,^18^ perovskite structures were predicted by first generating initial structures, and then searching for global minima via minima-hopping, utilizing on-the-fly generated classical potentials and DFT. Similarities between the two approaches are the heuristics for setting up the inorganic layer and placing the organic cations: Both methods try to place the cations onto similar “reference points”. The key difference is that the method presented in this work attempts to cover the configuration space purely by generating a wide variety of initial guesses (by widely sampling over molecule orientations), which are optimized independently as opposed to exploring the landscape through minima hopping (MH).

In general, we believe that RSS, MH, BH or combinations of different algorithms could work when used with the MACE model trained in this study. However, a proper assessment of the performance of these algorithms requires comparing the number of local minima visited before finding the global minimum, the length of MD simulations, number of external parameters, and sensitivity to these parameters. For performing new science, having a method that performs predictably across a diverse set of test cases, with little to no adjustable parameters, is also important. In this regard, we think that the constrained RSS approach in this work is attractive due to its simplicity and the fact that no decisions must be made before considering a new composition.

#### 3.3.2 Validation of the Model on Randomly Generated Structures

For an MLIP to be useful for the structure prediction task, the model must be accurate for the randomly generated structures and must not exhibit many spurious local minima. Crucially, it should reliably relax the structures to nearby DFT minima.

**Figure 6:**
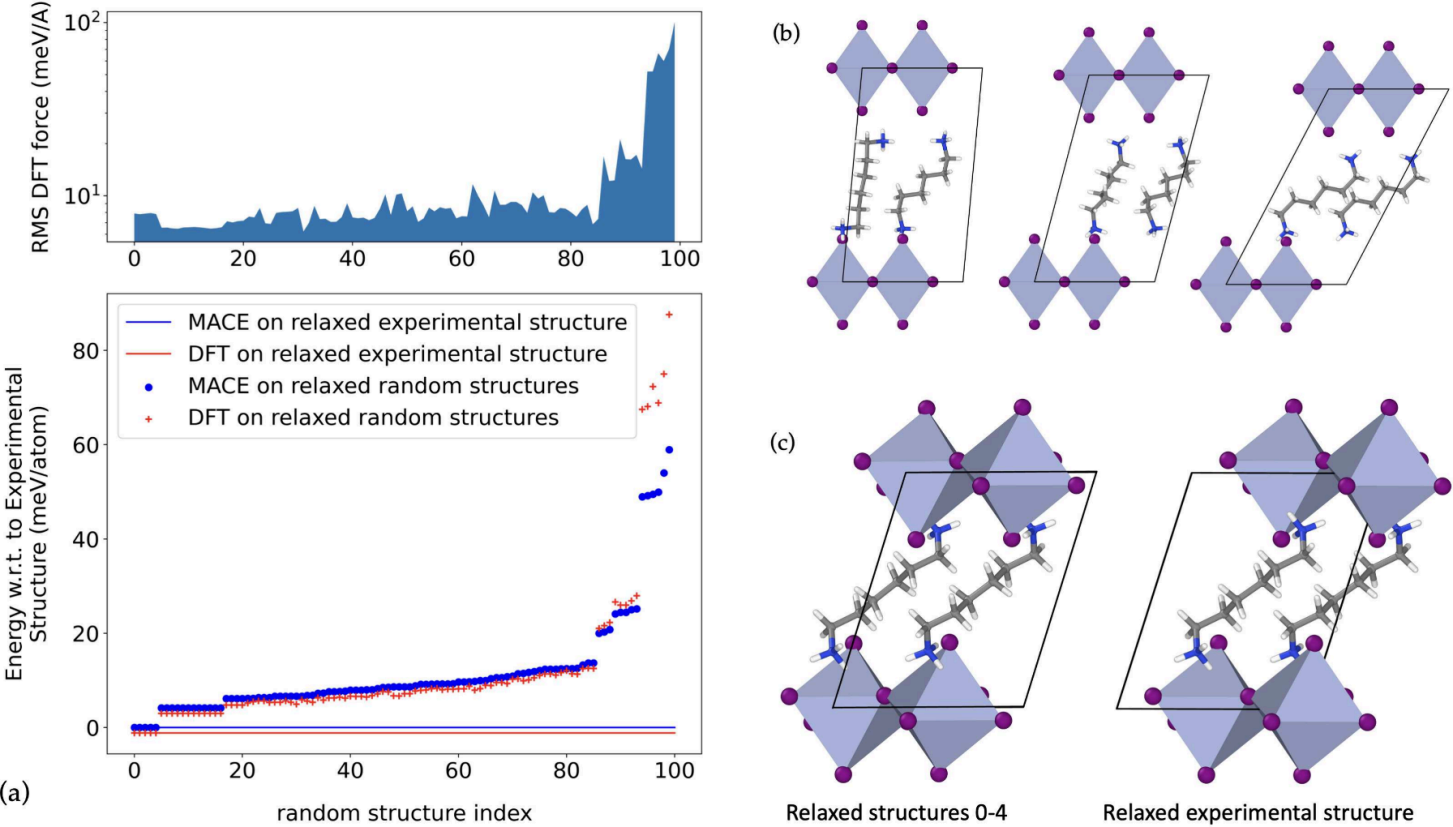
Rediscovering the structure of known a 2D perovskite. (a) Lower panel: formation energy (meV/atom) (blue) for the 100 randomly generated candidate structures, after geometry relaxation with the MLIP. Structures have been ordered according to increasing energy. Red points and lines show the same structures recalculated with DFT. Upper panel: Root mean square forces, according to DFT, of each of the relaxed structures. (b) Examples of the initial random configurations. (c) Comparison between the structure obtained by relaxing the experimentally determined structure, and the five lowest energy structures found by the screening method.

To demonstrate the accuracy of the model and structure searching method, we present the results of the process applied to a known 2D perovskite in Figure 6. Specifically, we take the perovskite formed by PbI_6_ octahedra in the inorganic layer and the organic cation NH_3_^+^[C]_6_NH_3_^+^. The (geometry relaxed) experimentally reported structure is shown in Figure 6c.

Given the composition, 100 random structures were generated by using the random generation procedure. Three examples of such structures are shown in Figure 6b. To simplify this demonstration, only the correct conformer of the organic cation was used to generate the samples. Subsequently, these 100 structures were relaxed using the MLIP. Since the initial samples are relatively high in energy, often containing a considerable amount of vacuum or nonphysical molecular arrangements, these geometry relaxations require several hundreds or even thousands of steps. Figure 6a shows the distribution of the energy of the resulting structures, ordered by increasing energy, relative to that of the experimentally reported structure. Also shown is the energy of the relaxed samples after re-evaluation with DFT.

Several important features can be noted. First, due to the nature of the long organic cation, which can stack in a variety of ways, the relaxation process reveals many local minima in the potential energy landscape. These appear as plateaus in the energy plot (bottom panel of Figure 6a). After recalculation of these structures with DFT, we see that the MLIP energy landscape is broadly correct in that these minima are correctly ordered with respect to DFT. The absolute energy error is also very low, being around 1 meV/atom which is roughly the accuracy of the model. Furthermore, the top of panel of Figure 6a shows the RMSE of forces, according to DFT, of the MLIP identified minima. For all but the highest energy configurations, the DFT forces are less than 10 meV/Å, suggesting that these are close to DFT minima.

The lowest energy structures identified by this procedure (the first five blue marks in Figure 6a) have an energy equal to that of the experimentally reported structure. This suggests that the process has indeed rediscovered the experimentally reported structure. This was confirmed by examining the five lowest energy relaxed structures. Up to rotations, reflections, and cell reductions, these structures are identical and match the experimentally reported structure as shown in Figure 6c.

In addition to the lowest energy structures, it should be emphasized that the method successfully captures other higher energy local minima.

The presence of these minima is responsible for the interesting behavior of 2D HOIPs at finite temperatures and external pressures.^5^ For single layer inorganic (*n* = 1) structures, dynamic disorder or ”melting” of the organics at elevated temperatures can lead to noticeable structural changes.^47,48^ Such phase transitions in 2D HOIPs are beyond the scope of this work, but we note that the presented model is likely to be accurate for higher temperature phases.

#### 3.3.3 Structure Prediction Performance across the Data Set

**Figure 7:**
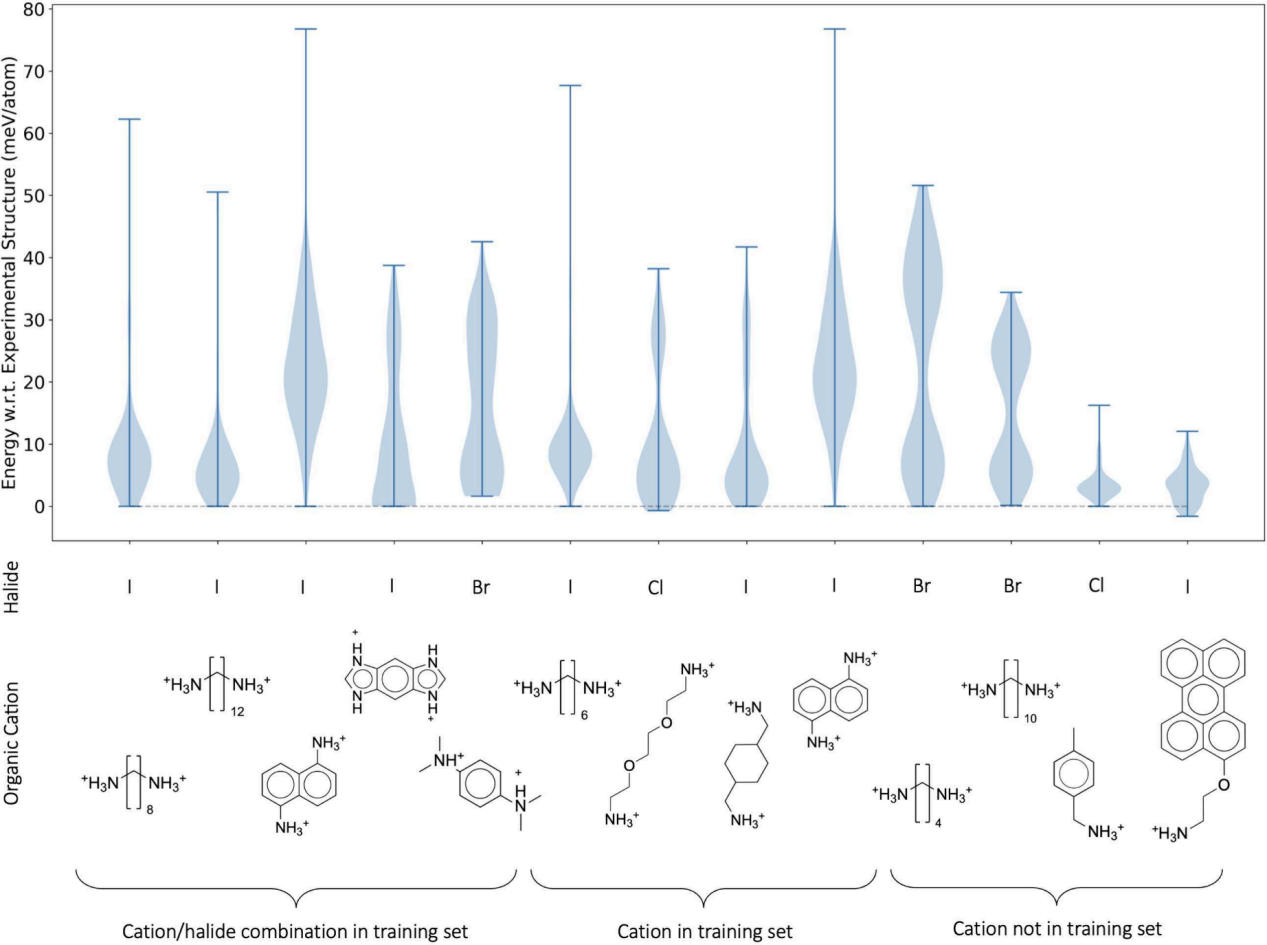
Performance of the RSS protocol applied to 13 experimentally known structures. Lower panel: Each combination of halide and organic cation shown in the lower part of the figure describes a perovskite present in the data set. Some of these structures were used to train the model whereas others are unseen. Upper panel: Violin plots of the energy distribution of the random samples after the MLIP geometry relaxation, relative to the energy of the geometry relaxed experimental structure. In the ideal case, the lower end of each violin plot would sit on the dashed line, indicating that the minimum energy structure found by the procedure is indeed the experimentally reported one. Structures are grouped into three categories. The first group contains perovskites which are present in the training set of the model. Following this are structures for which the combination of halide and cation is not present in the training set, but the cation is present paired with a different halide. The last group contains structures where the organic cation is not present anywhere in the training set.

We now demonstrate the usefulness of this procedure across a wider variety of 2D perovskites. The method described above has been applied to 13 structures in the data set. Figure 7 summarizes the results of this process. The lower rows identify the perovskite structure using the halide in the corner sharing octahedra of the inorganic layer and the organic cation. The upper panel shows the distribution of energies of the relaxed structures, following the random generation and relaxation process, with respect to that of the experimentally known structures. For this demonstration, 200 random structures were generated for each halide/cation combination. Only 200 samples were required since all but the last two structures in Figure 7 have unit cells containing only 2 organic cations.

Figure 7 also highlights which structures were present in the training set of the MLIP model. For the leftmost structures, samples of these perovskites acquired from molecular dynamics during the active learning process are present in the training set of our model. For the next set of structures, the organic cation is present somewhere in the data set, but the combination of cation and inorganic layer is not present. For the four right-most structures, the organic cation is not present in the data set.

In all but three cases, the identified structures with lowest energy correspond to the energy of the experimental structure. Subsequent comparisons showed that these structures did indeed match the experimentally reported version. Therefore, the combination of a simple RSS scheme with the developed MLIP can successfully identify the ground state structure of these complex systems.

In the three cases for which the lowest energy structure does not match the experimentally reported structure, one structure search failed to find any structures with an energy as low as that of the experimental structure within the 200 searches (the lowest energy found was about 2 meV/atom higher than the energy of the experimental structure). In the other cases, we confirmed that the procedure found the experimental minimum, as well as a lower energy structure. Subsequent evaluation with DFT revealed that these structures were also assigned lower energy than the experimental structure by DFT.

#### 3.3.4 Effect of Temperature on Crystal Structure

Metal–halide perovskites are known to exhibit phase transitions at accessible temperatures, which often occur via a tilting motion of the octahedra. The RSS process we have introduced, however, finds only the lowest energy, zero temperature phase. There are therefore some questions as to how far one can get before taking into account the finite temperature.

Since the RSS procedure typically finds the experimentally reported structure, we believe that, in most cases, the experiment probably probed the lowest energy phase, rather than those that would occur at higher temperatures. There is another possibility, which is that the RSS procedure favors wells in the PES which are “wide” and thus have greater entropy. When taking a finite number of samples, this might lead to some correlation between the RSS process missing the lowest energy state and the experiment also giving a higher energy state due to greater entropy at the experimental temperature.

One could examine the impact of the temperature by selecting a fixed number of low lying minima from each search and calculating a correction to the free energy. This could be done, for instance, from the phonon spectrum of the structure, which is easily computable using the MACE model. While this would be computationally quite cheap, since the simple RSS procedure has so far been successful in our experiments, we did not consider such corrections.

### 3.4 Prediction and Synthesis of New 2D Hybrid Perovskites

Finally, the structure search process was performed for a new organic cation with no previously known corresponding perovskite. Specifically, the combination of *cis*-1,3-cyclohexanediamine with a Pb–I inorganic layer was studied. This molecule is not present in our data set but consists of chemical groups which are well represented.

The structure searching procedure was conducted with a unit cell containing 8 copies of the organic cation across two layers. In total, 6000 samples were generated, with the large number being required due to the large number of molecules present in the unit cell. The perovskite was synthesized via slow hydrothermal growth and the resulting structure was determined, at 200 K, using a diffractometer as described in section 9.5.

**Figure 8:**
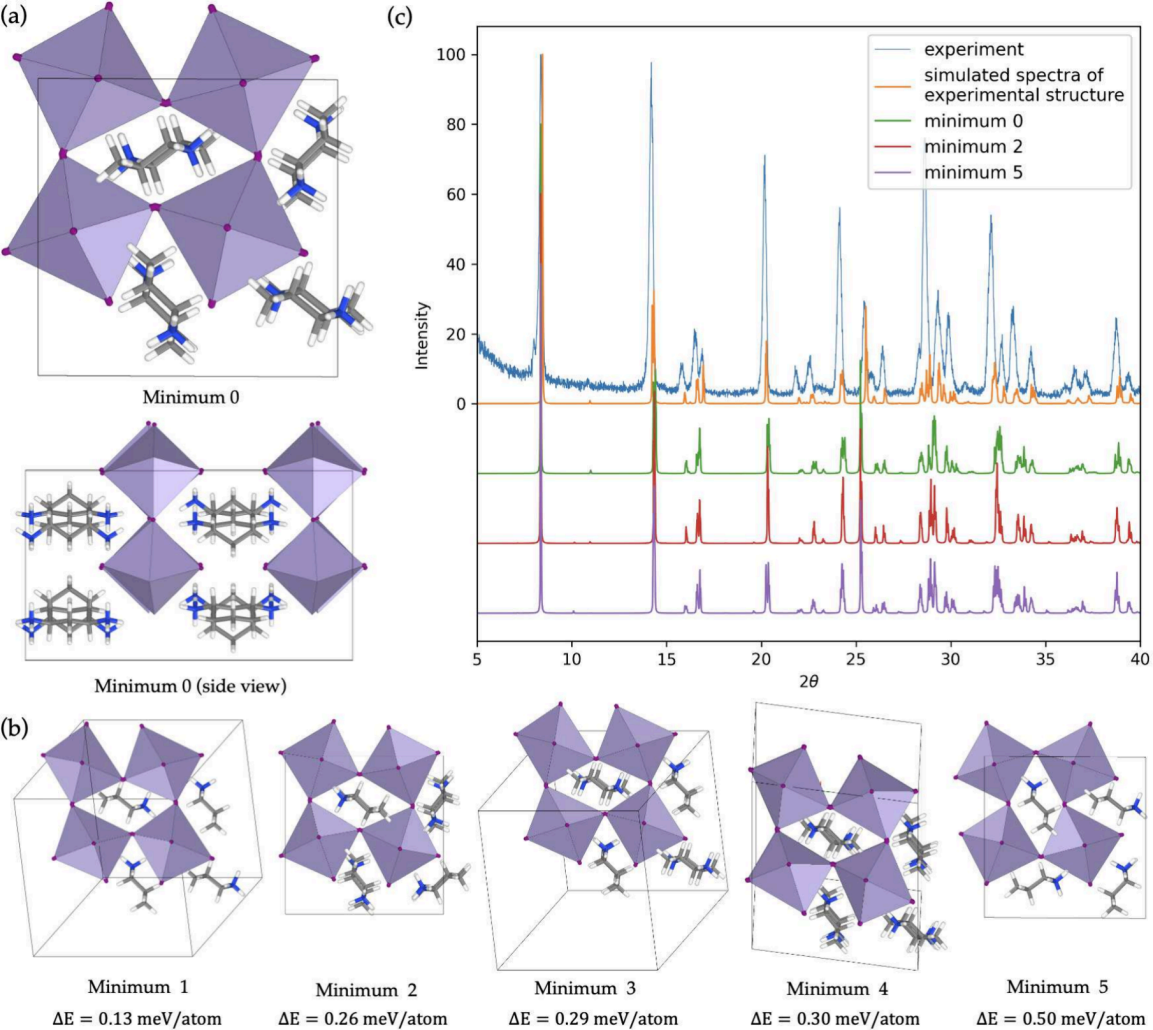
Comparing the lowest energy structures during the structure prediction task for a *cis*-1,3-cyclohexanediamine based perovskite. Since these unit cells are relatively complex, and differences between structures are subtle, we have tried to find “equivalent” representations of the unit cells for comparison. Cif files of all structures are available. (a) The lowest energy minimum found during our procedure, which is equivalent to the structure deduced by experiment. The unit cell contains 8 molecules and 232 atoms. (b) The next 5 lowest lying minima, and their energy above the lowest structure. All the structures shown contain 8 molecules, however when a structure adopts a higher symmetry and hence a smaller unit cell, some molecules appear to hide behind others. The key difference between minimum 0 and minimum 4 is the out of plane lattice vector. (c) Comparison of the experimentally measured pXRD with some chosen minima. The orange line labeled “simulated spectra of experimental structure” is the spectra as simulated by VESTA of the experimentally deduced unit cell.

Figure 8a and 8b show the resulting lowest energy structure (denoted as “minimum 0”), as well as the 5 next lowest energy structures that were predicted. As shown in the figure, the energy differences between the lowest lying minima are extremely small, with the 5 next best minima being only 0.5 meV/atom higher in energy than the ground state. This energy difference is smaller than the likely error in our model as well as the error of DFT due to finite k-point sampling.

Several interesting points can be made about these results: first, the lowest lying minimum found by the structure search process agrees with the experimentally measured structure. Since our model is fitted to DFT data which does not perfectly match the experiment, differences are unavoidable due to the error in the PBE functional for quantities such as equilibrium bond lengths. However, we can confirm that we predict the right structure by performing geometry relaxation with our MLIP of the experimentally reported structure. This resulted in exactly minimum 0, and the relaxation trajectory only involved only minor changes in bond length, as shown in the Supporting Information.

The 5 next lowest energy minima all involve similar orientations of the organic cation but differ in the set of reflections applied to the cations or the out of plane stacking vector. It is interesting to examine how easily one could differentiate between these structures using different experimental techniques. This was done by measuring the powder X-ray diffraction pattern (pXRD) of the synthesized HOIP, and comparing to the computed pattern of the lowest energy minima, as shown in Figure 8c. We compare the experimental result to the simulated spectra of minima 0, 2, and 5 as well as to the *simulated* spectra of experimentally deduced unit cell. The differences between the simulated pXRD of the experimentally reported structure and minimum 0 (orange and green in Figure 8c) come from only the aforementioned small differences in bond lengths. Interestingly, the spectra of the three numbered minima are almost indistinguishable; it would be extremely difficult to robustly differentiate these structures from the pXRD alone.

Furthermore, the small differences in molecular stacking lead to different optical properties. For example, out of the six structures in Figure 8, only a minimum of 0 is centrosymmetric. This means that it will not exhibit circular dichroism which is necessary for certain applications of 2D HOIPs. When targeting certain properties, a full picture of the landscape of low energy minimum is clearly important. Our structure searching method offers a window into this landscape, which could be used to choose experimental methods, or gain confidence in conclusions based on experimental results, in particular for properties that are strongly dependent on the structure.

Further predictions were made for 4 other organic cations which had no previously reported structure. These are discussed in Supporting Information section II.

### 3.5 Scalability and Computational Cost

Performing the above process requires geometry relaxation of many large crystal structures starting from high energy configurations. Typically, several hundred relaxations are required, with hundreds to thousands of dynamics steps for each relaxation.

In the structure searching process for the 13 structures in Figure 7, the average unit cell size was 78 atoms, and 200 samples were generated for each system. The entire set of calculations used to produce Figure 7 was performed in only 20 h on a single A100 GPU. This suggests that wide searches can be performed using modest computational resources. By comparison, a *single* DFT relaxation of one sample of the randomly generated structures shown in Figure 6 (similar in size and complexity to Figure 7 structures), performed on two nodes (256 cores) of AMD EPYC 7742, can take more than 1 day. Furthermore, the *N*^3^ scaling of DFT makes the same task for much larger systems infeasible.

One can also see the computational advantage of using our model in the structure search for the newly synthesized perovskite (section 3.4), which has 8 molecules and 232 atoms in the unit cell. For each sample of the 6000 generated structures, relaxation took between 2000 to 4000 steps, leading to a total computational cost of 240 GPU hours. We estimate that performing the same relaxations with DFT would require approximately 1.2 million CPU node-hours. In this case, the speedup corresponds to a factor of 10^4^. Note that the absolute times of course depend on the type of GPU and CPU hardware. Making a direct comparison not straightforward, but in terms of costing computational resources, an A100 GPU hour is approximately comparable to a node-hour with 128 CPU cores, and hence is the basis for the figures given above.

### 3.6 Extrapolation to Underrepresented Organics

**Figure 9:**
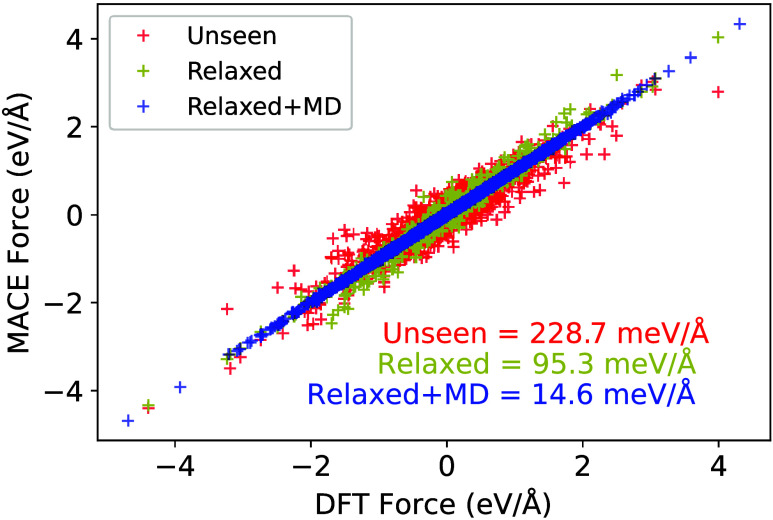
Force parity plot for cyclopropanaminium for three differently trained potentials. An “unseen” MLIP, with no samples of cyclopropanaminium, relaxed-model which has 200 randomly selected samples from the relaxation trajectories of the structure prediction model in the training set, and relaxed+MD which takes the top 10 most stable structures, followed by samples taken uniformly from MD trajectories. The two former MLIPs were trained independently.

We have demonstrated good performance of the trained MLIP both in terms of single point accuracy and in relaxing randomly generated HOIPs structures to global minima. However, it is the case that many of the organic molecules in the test set are structurally and chemically similar to the organics in the training set. Here we demonstrate an example of an organic cation in our test set, cyclopropanaminium (shown in Figure 9), for which the model performs poorly, and suggest an efficient way of retraining the model to improve the predictions.

For the original MLIP, which has not been trained on any examples of cyclopropyl alcohol, committee MD simulations immediately exceed the prescribed uncertainty threshold, indicating an uncertainty of the model in predicting forces. On samples that are taken from this MD, the model makes a large error with respect to DFT with a force RMSE of 228.7 meV/Å.

One approach is to add these uncertain high-error samples to the training set through AL to improve the MLIP for that specific organic. This is not possible when no experimental structure is known since an initial structure is needed for running the active learning. As shown in the Supporting Information Section III, we tested several approaches in which DFT calculations of only the isolated organic molecule were added to the training set, but these failed to improve the accuracy of the model to an acceptable level.

Another way of approaching this problem is to use the structure prediction algorithm to generate HOIP structures with the new organic cation. One can relax these candidates with the model, take samples from the relaxation trajectories, and add them to the training set. As shown in Figure 9, when the model is trained with 200 distinct samples from relaxation trajectories of cyclopropylaminium lead iodide, the resulting force RMSE is 95.3 meV/Å, a slight improvement over the original model. The meager improvement may be because many of the predicted relaxed structures are very similar, in terms of bond distances and orientation of the organic and inorganic components. A successful approach is to combine the structure prediction tool with MD simulations. Instead of taking 200 samples from the relaxations trajectories, we take only the 10 most stable structures predicted by the structure prediction algorithm, run short MD simulations (5 ps) and take samples uniformly every 1 ps from the MD trajectories. Note that we do not terminate the MD simulations based on uncertainty. Using this to add new data, the error in forces drops to 14.6 meV/Å, within the range of previously seen compositions in the training set. This is achieved with only a total of 50 new samples, and in one cycle of retraining.

This approach works because the original MLIP predicts physically reasonable structures, despite the large error in forces with respect to DFT. In particular, the model relaxes the randomly generated configurations to sensible structures in terms of the organic–inorganic stacking pattern. Similarly, MD simulations, while they may exhibit high committee uncertainty in forces, do not lead to unrealistic structures (e.g., no bond-breaking or coalescence of atoms).

## 4 Conclusions

We have presented an efficient, accurate, and general machine learning force field (MLIP) using the MACE architecture for lead based 2D HOIPs involving organic cations containing carbon, hydrogen, nitrogen, and oxygen. Our model performs well on single point energy and force predictions on samples taken from molecular dynamics simulations and can extrapolate to unseen organic cations.

Second, an RSS procedure has been presented with a set of heuristics designed to explore the landscape of these materials. These heuristics are relatively simple, with no adjustable parameters. Yet, the scheme covers the relevant space and is efficient in the total number of required samples. The combination of the RSS scheme and the MLIP is able to rediscover the experimentally reported structure for 13 2D HOIPs in our database. The model is demonstrably accurate during this process, correctly reproducing the complex energy landscape, as shown by exploring specific examples with DFT. The computational cost of our structure generation process and model is small enough that this procedure can be applied at scale.

Finally, our method was validated by synthesizing a new perovskite composition. Besides predicting the correct structure, the model revealed a delicate landscape of low-lying energy minima, which on its own, could be a useful investigative capability.

## 5 Methods

### 5.1 MACE Machine Learning Interatomic Potentials

This work has utilized the MACE framework for constructing the MLIP.^49^ MACE is a recently developed equivariant message passing tensor network that offers state of the art accuracy. The MACE architecture has been described and evaluated in detail previously.^37−40^ Therefore, the following description simply discusses some key aspects of the model design.

A MACE model predicts the total energy of a system as the sum of atom centered contributions. The environment around an atom is described by the atomic number and relative positions of neighboring atoms, up to some fixed cutoff: . The MACE architecture utilizes ideas from the atomic cluster expansion to efficiently construct atom centered features based on the local environment. These atom centered features are many-body, in that they depend simultaneously on atomic numbers and positions of several neighbors in a nontrivial way. These features are iteratively updated, and the final energetic contribution from each atom is expressed as a learnable function of these features.

The specifications of the MACE models used in this work are, in the nomenclature of reference,^39^ given in Table 2.

**Table 2:** Specifications of the MACE Models Used in This Study

number of chemical channels	128
maximum equivariance order L	1
single layer cutoff radius (Å)	5
number of layers	2

The loss function for MACE is
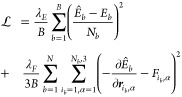
where *B* is the number of batches, *N*_*b*_ is the number of atoms in the batch, *E*_*b*_ is the DFT energy,  is the predicted energy, and  is the DFT force component in the direction α, of atom *i*_*b*_. *i*_*b*_ denotes the index within batch *b*. The λ_*F*_ and λ_*E*_ are the weights of the model which are set to 10 and 1000 respectively in the first 1500 epochs and then switched to 1000 and 1000 for the last 500 epochs.

### 5.2 Molecular-Dynamics and Geometry Relaxations with MACE Potentials

All molecular dynamics (MD) simulations were carried out using the atomic simulation environment (ASE) package^50^ in the NPT ensemble at 300 K and 1 atm. A Nosé–Hoover thermostat^51,52^ was used. During active learning, MD simulations were propagated using the average prediction of 3 committee members. The relative force uncertainty *f*_*rel*_^*i*^ is defined as
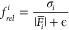
1where σ_*i*_ and  denote the standard deviation and mean of forces over the committee members on atom *i*. *ϵ* is a regularizer to avoid diverging ratios for small forces. At each MD step, the atom with the greatest *f*_*rel*_^*i*^ is selected, and this value is compared against the predefined threshold of 0.2. If this uncertainty indicator exceeds the threshold, the simulation is terminated. The regularizer *ϵ* for all of the simulations was set to 0.2 eV/Å.

All geometry relaxations have been done using preconditioned LBFGS as the optimizer.^53^ During relaxations, both cell sizes and atomic positions are allowed to change, and the relaxed cell is achieved when the maximum force on each atom is less than 1 meV/Å.

### 5.3 Electronic Structure Calculations

All the electronic structure calculations for either relaxation or single point calculations are performed using Vienna Ab initio Simulations Package (VASP)^54,55^ with the PBE^56^ for the exchange-correlation functional and the projector augmented-wave (PAW) pseudopotentials.^57,58^ Dispersion energy-corrections are applied using D3 approximation.^59^ All calculations used a Γ-centered Monkhorst–Pack^60^ k-point mesh with a density of 1000 k-points per number of atoms scaled proportionally to the length of the reciprocal lattice vectors, as implemented in pymatgen.^61^ The electronic wave functions were expanded in a plane wave basis set with an energy cutoff of 600 eV.

### 5.4 An Algorithm for Random Structure Generation of 2D HOIPs

A random structure generation algorithm was developed to demonstrate the usefulness of the MACE model. Our algorithm is not intended to be completely general and relies on several simplifications. Future developments could utilize methods from organic crystal structure prediction for more generality. The code used in this project is available as a python package at https://github.com/WillBaldwin0/LDHP-builder.

The procedure is as follows: The inorganic layer is first generated from lead and the chosen halide as a monolayer of regular lead–hailde octahedra. The lead–halide bond length is chosen to be the average of such bonds across our training set.

For each organic cation to be placed in the unit cell, the following process is performed. We assume that the molecule joins to the layer in a certain way: Salient points on the molecule are defined as the heavy atoms on the “extremities”. In practice, this is done by first finding the moment of inertia tensor of the molecule and interpreting the eigenvectors as a local coordinate basis for the molecule. For molecules that are longest in a certain direction, the eigenvector with the smallest eigenvalue is generally directed along this direction. The extremities of the molecule are defined as the heavy atoms that have the largest relative distance between one another when projected onto this vector. One of these heavy atoms serves as a reference atom, which is placed onto a given point on the inorganic layer. The orientation of the cation is then determined by first applying a random rotation about the selected atom and subsequently applying up to two reflections in planes normal to the lattice vectors of the inorganic monolayer. Only one rotation vector is chosen for all the molecules, but since reflections are applied afterward, this still spans a wide range of molecular stacking patterns.

We also employ a heuristic when assigning reflections to molecules. Given a unit cell that contains *N* = 2^*p*^ molecules, the reflections in the *x*-axis (assuming *z* is the out-of-plane direction) are encoded via a binary array . *x*_*n*_ = 1 means that the *n*’th molecule is reflected, whereas *x*_*n*_ = 0 means it is not. While one could randomly choose a binary array of length *N*, we found that a good heuristic was to instead sample only arrays with the following form:
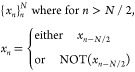


The choice to copy *x*_*n*–*N*/2_ or negate it is the same for all *n* = 1, ..., *N*/2. The same is done for reflections on the *y*-axis. This naturally encodes some symmetry into the molecular orientation and improved efficiency when working with many molecules in the unit cell.

After the orientation of all molecules is determined, the molecules are checked for intersections. Assuming there are no intersections, the out of plane lattice constant is fixed to remove as much vacuum from the cell as possible. The result of this procedure is a monolayer with a set of organic cations at random orientations on the layer. See Figure 6b for example structures from this procedure.

### 5.5 Experimental Synthesis and Characterization

The perovskite 1,3-(*cis*)-cyclohexanediamine-PbI_4_ was synthesized in order to compare the observed crystal structure with the results obtained via the computational methods described previously. Crystals of the perovskite were obtained through slow hydrothermal growth by dissolving equimolar amounts of the amine and lead(II) iodide in concentrated hydriodic acid in a sealed pressure vessel at 150 °C and cooled at a rate of 5 °C/h, resulting in the formation of millimeter-scale orange crystalline chunks. Residual hydriodic acid was removed by washing with methylene chloride and diethyl ether, followed by drying under a vacuum for several days. The crystal pieces are highly stable in an ambient atmosphere and demonstrate no signs of decomposition over weeks of storage.

The crystal structure was determined by using a Rigaku XtaLAB Synergy diffractometer. Crystal samples were mounted in oil on a ring-loop and placed in a cryo N_2_ stream at 200 K. CrysAlis Pro was used to screen and collect diffraction patterns using Mo K_α_ (λ = 0.71073 Å). A full sphere of diffraction data was collected, and a multiscan empirical absorption correction was applied. The maximum resolution that was achieved was Θ = 31.00° (0.69 Å). The structures were solved with the ShelXT structure solution program using the Intrinsic Phasing solution method and by using **Olex2** as the graphical interface. The model was refined with version 2016/6 of ShelXL 2016/6 using Least Squares minimization. The crystal structure was determined with minimal guidance beyond initial atomic assignment, and the resulting solved structure featured a low *R* value indicating that the solved structure aligned well with the atomic positions observed in the diffraction pattern.

Powder XRD (pXRD) was measured on a Rigaku SmartLab as an additional point of comparison between both the predicted and experimental crystal structures to assess the presence of any additional crystal phases at room temperature that may contribute to different structural behavior. Samples were prepared from the as-grown ABX_4_ perovskite crystals by grinding in a mortar and pestle to ensure uniform distribution of the powder particle size and orientation. All measurements were performed at room temperature under ambient atmosphere. The θ/2θ spectra of the perovskite powders were then compared to predicted patterns generated from either the experimental or as-modeled crystal structures.

Simulations of pXRD were performed in the VESTA structure visualization software package.^62^

## Data Availability

The committee of MACE models trained on the full training set is available in a zenodo repository 10.5281/zenodo.10729400. The full train and test sets are also available as a python pandas dataframe. The experimentally determined newly synthesized structure, as well as the five predicted lowest energy structures found by our process, are also available. The random structure generation algorithm was implemented in a python package which can be found at https://github.com/WillBaldwin0/LDHP-builder.

## Supporting Information Available

Structural comparison between synthesized and predicted structures; Extrapolation to 1D HOIPs; Detailed analysis of underrepresented molecules (PDF)
