# Supplementary material for: Accurate Crystal Structure Prediction of New 2D Hybrid Organic–Inorganic Perovskites
Source: J Am Chem Soc. 2024 Sep 30;146(40):27392–404. doi: 10.1021/jacs.4c06549 (PMC11468779; doi:10.1021/jacs.4c06549)
Supplement: Supplementary file 1 — ja4c06549_si_001.pdf [file ja4c06549_si_001.pdf]

Supporting Information:
Accurate Crystal Structure Prediction of New 2D Hybrid Organic Inorganic Perovskites

Nima Karimitari,[1,*] William J. Baldwin,[2,†,*] Evan W. Muller,[3] Zachary J. L. Bare,[1] W. Joshua Kennedy,[4] Gábor Csányi,[2] and Christopher Sutton[1,‡]

[1]*Department of Chemistry and Biochemistry, University of South Carolina, South Carolina 29208, United States*
[2]*Department of Engineering, University of Cambridge, Cambridge CB2 1PZ, UK*
[3]*UES, Inc., Beavercreek, Ohio 45432, United States*
[4]*Materials and Manufacturing Directorate, Air Force Research Laboratory, Wright-Patterson AFB, Dayton, Ohio 45433, United States*

(Dated: July 16, 2024)

I. PREDICTIONS FOR THE NEWLY SYNTHESISED PEROVSKITE

When validating our prediction of the newly synthesised perovskite, numerical comparisons are similar but do not perfectly match since our model is trained to reproduce the energy landscape of DFT calculations with PBE+D3 functional. Therefore, there is an overestimation of 3-5% in bond length distances compared to the experiment, as expected from PBE+D3 functionals. We confirm our prediction is correct by firstly examining the two structures by eye, which are shown in Fig. 1. Secondly, we showed that a geometry relaxation of the experimentally reported structure with our model results in exactly the predicted structure. Even though the figure shows that this does not meaningfully change the structure, this is the origin of the differences between the predicted pXRD of the experimentally deduced structure and our prediction in the main text Fig. 8.

The computational cost of the structure prediction algorithm is highly dependent on the number of molecules in the unit cell since this dictates the number of samples required. In our dataset, the median number of molecules per unit cell (for perovskites with no locally 3D regions) was 4. Evidently there is a trade-off between running fewer, expensive, searches with large unit cells, or many cheaper searches with small cells and accepting that one will occasionally miss the correct structure.

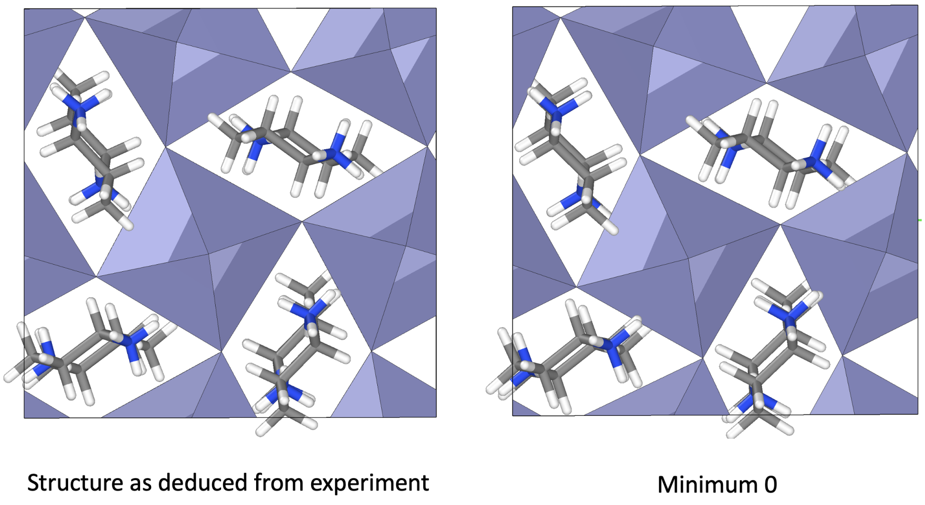

FIG. 1: Comparing the structure as deduced from experiment to the lowest minima found by our process.

*These authors contributed equally
†Electronic address: wjb48@cam.ac.uk
‡Electronic address: cs113@mailbox.sc.edu

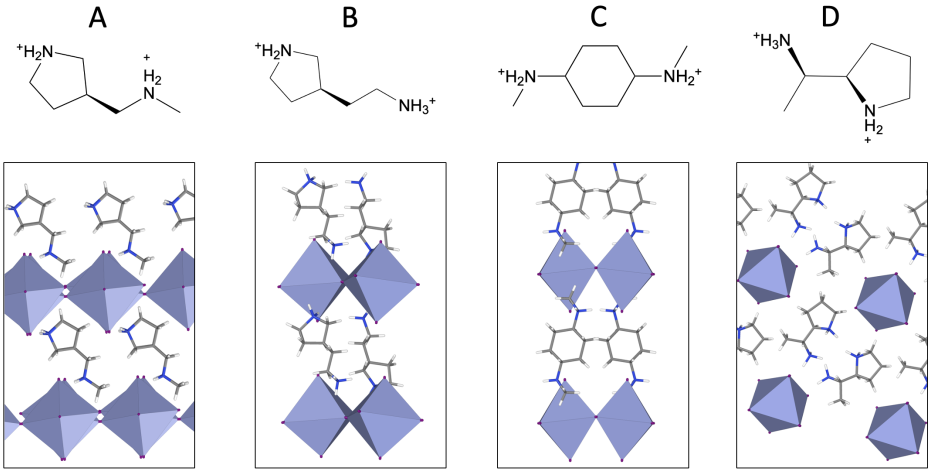

FIG. 2: Applying our random structure search procedure to 4 molecules with chiral centers. The lowest energy structure found for the right-most molecule relaxed to a 1D perovskite.

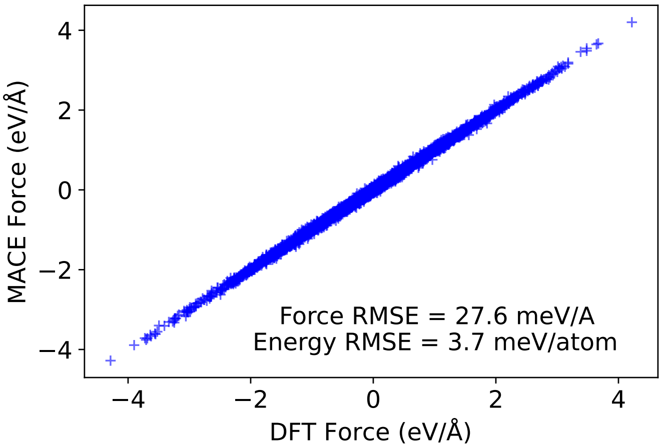

FIG. 3: The force parity plot between the model and DFT on samples taken from an MD simulations of predicted 1D HOIP.

II. EXTRAPOLATION TO 1D PEROVSKITES

While conducting random structure searches of potential new HOIPs, it was discovered that the final MACE model is able to extrapolate well to 1D HOIPs, without having seen any in the training set.

We applied the random structure search procedure to four organic cations with chiral centers, as shown in Fig. 2 and predicted the most stable HOIPs that they can form. These structure searches were performed with a small unit cell, containing just 2 molecules and one inorganic layer. An interesting case is molecule D in Fig. 2, 2-(1-aminoethyl)pyrrolidinium, for which the most stable structure is a 1D HOIP. This is surprising because the structure search procedure was the same as described in the main text, where the initial generated samples are all 2D layered PbX_4 structures, and the model was able to achieve a structure with PbX_3 inorganic unit (the additional halide ions are isolated, away from the rest of the inorganic framework). To examine the accuracy of the model and stability of this structure, a constant pressure MD simulation of the structure was ran at 300 K, 1 atm for 50 ps. The maximum relative force uncertainty remains below the threshold (as discussed in main text section II) at all times. Furthermore, the root mean square error with respect to DFT (see also Fig. 3), for samples taken every 5 ps was 27.5 meV/Åand 3.7 meV/atom for forces and energy, respectively. This close agreement with DFT implies that the current trained model, which has not trained to any lower dimensional HOIPs, is accurate in dealing with 1D HOIPs.

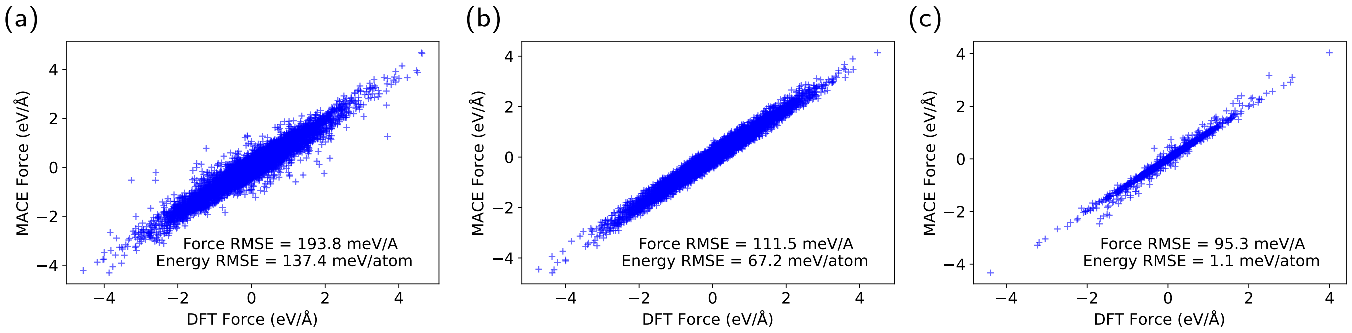

FIG. 4: Force parity plots for three different analysis of extrapolating to underrepresented molecules. All are taken from independent MD simulations with (a) trained to isolated molecules in box, (b) trained to only the forces of the isolated molecules, and (c) trained to the samples taken from the relaxation trajectory.

III. UNDERREPRESENTED ORGANICS

One of the challenges of the trained potential is in dealing with organic molecules with local structural features and functional groups that are very different to those found in the training dataset. One example, as discussed in the main text Section V is cyclopropanaminium. Several other approaches were tested while searching for ways to quickly add new information about unseen organic molecules. Some of these methods all involved performing calculations of isolated molecules, and adding just these to the training set. Here we summarise out the outcomes of these analyses.

(a) **Isolated Molecules.** We place the isolated molecule in an empty box ($15 \times 15 \times 15$ Å) and ran collected samples by running molecular dynamics simulations using extended tight binding (xTB)[1]. Specifically, the GFN2-xTB[2] was used via the xtb-python api. Simulations were ran at 500K using a time step of 1 fs and 200 samples were taken at intervals of 1 ps. These configurations were subsequently evaluated with DFT using the same settings as the perovskite training data. One difficulty with this method is that cations in HOIPs are charged molecules and ignoring the charge in the isolated molecule calculations can severally affect the force/energy errors. However, including them is also a problem due to long range interactions between neighboring unitcells. We considered the charged cations but with the dipole and quadropole corrections [3]. The original model retrained to this dataset shows higher than expect errors as shown in Fig. 4a. The retrained model have a very poor prediction for energies. This is because even with the presence of corrections to long-range interactions, the energy difference between charged and uncharged calculations is so large that the current MLIP, trained to cations that are present in the periodic systems, cannot accurately predict both isolated molecules and molecules in solids.

(b) **Isolated Molecules, Force Information Only.** As a final test on the isolated molecules, we then tried to train the original model to only forces of the isolated cyclopropanaminium. This modification led to significant improvement in both force and energy predictions, as shown in Fig. 4b, but still the error was an order of magnitude larger than the values reported for seen perovskites in Section II.B.

(c) **Structures found by Random Structure Search.** Instead of dealing with the isolated molecules, we used the random structure search procedure to generate initial HOIPs with this molecule and then relax them with the MLIP. While the MLIP used in the structure search struggles with energy and forces, it can still create samples that are useful for retraining the model. In Fig. 4c, we retrained the original model to 200 randomly selected samples from the relaxation trajectories. This leads to an improvement with errors of 95.3 meV/Åand 1.1 meV/atom for forces and energy.

(d) **Random Structure Search + MD.** In our final experiment, we combined the previous step with MD simulations. This is because in relaxation trajectories, many of the samples are structurally similar making the learning inefficient, but MD simulations can lead to more diverse local environments that the model can learn from. Therefore, we collected the 5 most stable structures predicted by the search algorithm method, ran MD simulations for 10 ps. In total 50 samples (taken every 1 ps) are then added to the original training set and the model is retrained. The errors are at 14.6 meV/Åand 0.9 meV/atom also shown in main text in Sec. V.

[1] C. Bannwarth, E. Caldeweyher, S. Ehlert, A. Hansen, P. Pracht, J. Seibert, S. Spicher, and S. Grimme, "Extended tight-binding quantum chemistry methods," *WIREs Computational Molecular Science*, vol. 11, no. 2, p. e1493, 2021. [Online]. Available: https://wires.onlinelibrary.wiley.com/doi/abs/10.1002/wcms.1493

[2] C. Bannwarth, S. Ehlert, and S. Grimme, "Gfn2-xtb—an accurate and broadly parametrized self-consistent tight-binding quantum chemical method with multipole electrostatics and density-dependent dispersion contributions," *Journal of Chemical Theory and Computation*, vol. 15, no. 3, pp. 1652–1671, 2019, pMID: 30741547. [Online]. Available: https://doi.org/10.1021/acs.jctc.8b01176

[3] J. Neugebauer and M. Scheffler, "Adsorbate-substrate and adsorbate-adsorbate interactions of na and k adlayers on al(111)," *Phys. Rev. B*, vol. 46, pp. 16 067–16 080, Dec 1992. [Online]. Available: https://link.aps.org/doi/10.1103/PhysRevB.46.16067
